# Interspecies signaling modulates the biosynthesis of antimicrobial secondary metabolites related to biological control activities of *Pseudomonas fluorescens* 2P24

**DOI:** 10.1128/spectrum.01886-24

**Published:** 2025-02-03

**Authors:** Nannan Zhang, Xianfeng Zhu, Xuanying Tao, Jie Li, Qi Tang, Xiaochun Liu, Li-Ming Luo, Pingping Zhang, Li-Qun Zhang, Yong-Xing He, Honghua Ge

**Affiliations:** 1 School of Life Sciences, Anhui University, Hefei, China; 2 Institute of Health Sciences and Technology, Anhui University, Hefei, China; 3 Ministry of Education Key Laboratory of Cell Activities and Stress Adaptations, School of Life Sciences, Lanzhou University, Lanzhou, China; 4 School of Veterinary Medicine and Biosecurity, Lanzhou University, Lanzhou, China; 5 College of Plant Protection, China Agricultural University, Beijing, China; University of Mississippi, University, Mississippi, USA

**Keywords:** *Pseudomonas*, pyoluteorin, interspecies signaling, transcriptional repressor, 2,4-diacetylphloroglucinol

## Abstract

Signaling between rhizosphere microorganisms is crucial in bacteria interaction and communication, shaping the rhizomicrobiome. Plant growth-promoting bacterium *Pseudomonas* produces a spectrum of important antibiotics to inhibit plant pathogens, albeit with an associated metabolic burden. Antibiotics could function as intra- and interspecies signals at subinhibitory concentrations to coordinate gene expression and microbial behaviors. In this work, we characterized pyoluteorin as an interspecies signal that modulates the biosynthesis of 2,4-diacetylphloroglucinol (2,4-DAPG), a broad-spectrum biocontrol agent, in non-pyoluteorin-producing *Pseudomonas fluorescens* 2P24. We demonstrated that the key transcriptional repressor PhlF from the 2,4-DAPG biosynthetic gene cluster spontaneously senses pyoluteorin, enhancing repression of the *phlA* promoter activity and inhibiting 2,4-DAPG synthesis in *P. fluorescens* 2P24. Pyoluteorin also binds to another transcriptional repressor, PhlH, from the 2,4-DAPG biosynthetic gene cluster, subsequently releasing the transcription of *phlG*, which facilitates the hydrolysis of 2,4-DAPG. Both PhlF and PhlH are simultaneously involved in sensing exogenous pyoluteorin to regulate the 2,4-DAPG biosynthetic operon, playing a crucial role in controlling antibiotic metabolites in response to environmental changes. Further phylogenetic and structural analyses demonstrated that PhlH and PhlF are widely distributed across *Pseudomonas* spp. with conserved ligand-binding domains. The findings shed new light on the regulatory mechanism of 2,4-DAPG biosynthesis underlying interspecies signaling by pyoluteorin and provide invaluable clues for the rational design of co-inhabiting *Pseudomonas* spp. as biocontrol agents.

**IMPORTANCE:**

Rhizosphere microorganisms release vital signals that shape microbial communities, with antibiotics at low concentrations acting as intra- and interspecies signals. However, the mechanisms of these signals in coordinating gene expression are unclear. In non-pyoluteorin-producing *Pseudomonas fluorescens* 2P24, pyoluteorin was identified as an interspecies signal that regulates the *phl* biosynthesis gene cluster for 2,4-DAPG production. TetR family repressors PhlH and PhlF were found to positively regulate 2,4-DAPG hydrolysis and negatively regulate its synthesis in response to pyoluteorin. Structural modeling and docking analyses revealed the interactions between pyoluteorin and both PhlH and PhlF, modulating gene expression. Phylogenetic analyses showed a wide distribution of PhlH and PhlF across *Pseudomonas* spp. with conserved ligand-binding domains. These findings deepen our understanding of interspecies signaling mechanisms and highlight the potential for designing co-inhabiting *Pseudomonas* spp. as effective biocontrol agents.

## INTRODUCTION

Rhizosphere microorganisms, known as plant-associated microbial communities, are composed of diverse bacteria affecting plant development (1). Microorganisms in the rhizosphere interact and communicate with one another, playing a pivotal role in the ecological fitness of host plants (2). Signaling among microorganisms in the rhizosphere involves a common class of molecular signals, including quorum-sensing signals acyl-homoserine lactone and diffusible signal factors (3), volatile organic compounds (4), diketopiperazines (5), and antibiotics at subinhibitory concentrations (6). These signaling molecules are essential for modifying and shaping microbial communities (2). Certain antibiotics can function as intra- and interspecies signaling molecules at low and non-inhibitory concentrations, most often associated with coordinating gene expression and microbial behaviors (7).

*Pseudomonas* is a predominant group of plant growth-promoting bacteria that produce a spectrum of important agricultural antibiotics with antagonistic activities to suppress plant pathogens (8). Pyoluteorin (PLT) and 2,4-diacetylphloroglucinol (2,4-DAPG), the two polyketide metabolites produced by *Pseudomonas* spp., are broad-spectrum antibiotics with antibacterial and antifungal activities (9). PLT is synthesized by a hybrid non-ribosomal peptide synthetase and polyketide synthase gene cluster and exhibits excellent antagonistic activity against the oomycete, including the plant pathogen *Pythium ultimum* (10). A type III polyketide synthase gene cluster is responsible for synthesizing 2,4-DAPG that contributes to the inhibition of fungal pathogens. Within the typical *phl* gene cluster of 2,4-DAPG, two pairs of oppositely transcribed operons, namely, *phlF*–*phlACBD* and *phlG*–*phlH*, are integral to the biosynthesis of 2,4-DAPG (11). The *phlD* gene encodes type III polyketide synthase responsible for converting malonyl-CoA into PG, which is further transformed into monoacetylphloroglucinol (MAPG) and 2,4-DAPG by acetyltransferase PhlACB through a series of enzymatic steps (12). Meanwhile, *phlG* encodes a hydrolase enzyme that degrades 2,4-DAPG into MAPG (13). Many *Pseudomonas protegens* strains possess both PLT and 2,4-DAPG biosynthetic gene clusters, such as *Pseudomonas protegens* Pf-5, CHA0, H78*,* FD6*,* and MP12, while certain *Pseudomonas* strains produce either PLT or 2,4-DAPG exclusively (14–17). PLT-producing *Pseudomonas* strains, while encompassing some strains of *Pseudomonas aeruginosa* and *Pseudomonas* sp. M18, typically do not produce 2,4-DAPG (18, 19). On the other hand, certain strains of *Pseudomonas* spp., such as *Pseudomonas aureofaciens* Q2-87, *Pseudomonas fluorescens* F113, and *Pseudomonas fluorescens* 2P24, synthesize 2,4-DAPG but not PLT (15, 20).

PLT acts as an endogenous signal and autoinduces the activation of the LysR-type regulator PltR and TetR-type regulator PltZ, which positively and negatively regulate the *plt* gene cluster, respectively (21). Similarly, 2,4-DAPG is recognized by TetR family repressors and PhlF of the 2,4-DAPG biosynthetic operon. 2,4-DAPG dissociates the repressor PhlF from the *phlACBD* operon to activate transcription (22). PhlH recognizes 2,4-DAPG to derepress the expression of PhlG and decrease the production of 2,4-DAPG (23). In addition to the autoregulation of PLT and 2,4-DAPG, there is evidence of cross-regulation between these compounds in the signaling pathway. Phloroglucinol (PG), an intermediate for the synthesis of 2,4-DAPG, is converted into chlorinated derivative PG-Cl and PG-Cl_2_ by halogenases encoded by *pltM* in the *plt* gene cluster from *P. protegen* Pf-5. These chlorinated derivatives function as an intraspecies signal regulating PltR to induce the transcription *plt* gene cluster (14, 24). Recent studies indicate that PLT negatively regulates the biosynthesis of 2,4-DAPG via PhlH in *P. protegen* Pf-5 (25). It elucidates that PLT as an intraspecies signal is crucial for the cross-regulation between the biosynthesis pathways of PLT and 2,4-DAPG within a single *Pseudomonas* strain. The interspecies competition between *P. protegens* and *Pseudomonas capeferrum* reveals that PLT produced by *P. protegens* can be part of intraspecies signaling, resulting in the subsequent repression of 2,4-DAPG biosynthetic gene clusters and cell lysis of *P. capeferrum* (26).

Rhizosphere microorganisms release vital signals that shape microbial communities, with antibiotics at low concentrations acting as intra- and interspecies signals. However, the mechanisms of these signals in coordinating gene expression are largely unclear. Investigating the potential of PLT as an interspecies signal is important for understanding the interspecies communications in *Pseudomonas* and its impact on biocontrol activity. Our current work demonstrates that non-PLT-producing *P. fluorescens* 2P24 senses subinhibitory concentrations of exogenous PLT to down-regulate the production of 2,4-DAPG via the TetR family repressors PhlH and PhlF. Pyoluteorin was identified as an interspecies signal in *P. fluorescens* 2P24, regulating the *phl* biosynthetic gene cluster for 2,4-DAPG production. TetR repressors PhlH and PhlF positively regulate 2,4-DAPG hydrolysis and negatively regulate its synthesis in response to pyoluteorin. Docking and structural analyses revealed the interaction of pyoluteorin with PhlH and PhlF, modulating gene expression. Phylogenetic analysis showed a wide distribution of PhlH and PhlF across *Pseudomonas* spp. with conserved ligand-binding domains (LBDs). These findings enhance understanding of the PLT-mediated interspecies signaling pathway, with implications for designing co-inhabiting *Pseudomonas* spp. as effective biocontrol agents.

## MATERIALS AND METHODS

### Bacterial strains, plasmids, and culture conditions

The bacterial strains, plasmids, and primers used in this study are listed in Tables S1 and S2, respectively. *P. fluorescens* 2P24 and its derivatives were grown at 28°C at 200 rpm in liquid King’s B (KB) medium or agar plates. *Escherichia coli* and its derivatives were grown at 37°C at 200 rpm in Luria–Bertani (LB) broth or agar plates. When necessary, the bacterial growth media were supplemented with ampicillin (50 mg/L), kanamycin sulfate (30 mg/L), chloramphenicol (100 mg/L), sucrose (10%) (wt/vol), and isopropyl β-D-1-thiogalactopyranoside (0.2 mM).

### Cell growth assay

All strains were grown to stationary phase in LB medium and then diluted in KB medium. The initial turbidity of each *Pseudomonas fluorescens* 2P24 culture was adjusted to an OD_600_ of 0.1. Each culture was grown in 100 mL of KB medium at 28°C. The cultures were treated with either 0 µM (control) or 20 or 40 µM of PLT. All cultures were incubated at 28°C for 50 h, and growth was monitored by measuring OD_600_ at regular intervals. The growth curves were recorded at 600 nm by a multimode reader. Three independent experiments were performed, and the error bars were calculated based on the data.

### Construction of *P. fluorescens* 2P24 mutants and derivative strains

A two-step homologous recombination method was used to construct an in-frame deletion mutant of *P. fluorescens* 2P24. The DNA fragments containing both flanking sequences (~1 kb) of target genes were amplified by PCR using primers in Table S1. The DNA fragments were cloned into the suicide vector pK18mobsacB-Km. The resulting plasmids were conjugated into the 2P24 strain with the help of the donor *E. coli* S17-1 strain on the KB plate. After two homologous recombination and selection with high concentrations of sucrose, the wild-type copy was replaced by the deleted version. The deleted version replaced the wild type after two recombination events under high-sucrose stress. All the deletion mutants were confirmed by PCR amplification and sequencing.

### Quantification of 2,4-DAPG

The production of 2,4-DAPG was quantified using high-performance liquid chromatography (HPLC) following the method described by Bonsall et al. (27). *P. fluorescens* 2P24 was inoculated at a starting OD_600_ of 0.02 in 50 mL King’s B medium. After 20, 28, and 34 h of the fermentation, with and without 20 μM PLT, 1 mL of culture broth was collected and centrifuged at 12,000 rpm for 10 min. The supernatant was filtered through a 0.22 µm mixed cellulose ester syringe filter and further analyzed by high-performance liquid chromatography (LC-2030plus, SHIMADZU, Japan) equipped with WondaSil C18-WR column (5 µm, 4.6 × 150 mm). Quantification of 2,4-DAPG was performed using a water/acetonitrile gradient (5%–100% acetonitrile with 0.1% trifluoroacetic acid) over 15 min at a wavelength of 270 nm, with a flow rate of 1 mL/min. The column was pre-equilibrated with 5% acetonitrile for 1 h. The composition of the mobile phase was changed by a linear gradient mode. The gradient was performed from 5% to 40% acetonitrile in 4.5 min, from 40% to 100% acetonitrile in 4.5 min, and then from 100% to 0% acetonitrile in 4 min, at a flow rate of 1.0 mL/min. The retention time was 8.9 min for 2,4-DAPG. Quantitation was based on a standard curve prepared using a chemical standard of 2,4-DAPG (Sigma-Aldrich).

### Total RNA extraction, cDNA preparation, and quantitative real-time PCR assay

Total RNA was extracted by the TRIzol method. Samples were harvested by centrifugation at low temperatures, and then total RNA was extracted by TRIzol and chloroform. The cDNAs were synthesized by using RT-PCR Kit (HiScript III RT SuperMix for qPCR, Vazyme). For real-time PCR experiments, each PCR reaction (20 μL) contained 10 μL of AceQ Universal SYBR qPCR Master Mix (Vazyme), 2 μL of cDNA samples, and 0.2 μM primers. The reactions were performed in a QuantStudio 6 Flex Real-Time PCR System. Quantitative analysis of the initial template was achieved by real-time monitoring of the fluorescence signal changes during the PCR reaction. The 16S rRNA gene was set as an internal reference to normalize the data, and The −ΔΔCT method was used for relative quantification. Three independent experiments were performed, and the error bars were calculated.

### Protein expression and purification

The recombinant protein of PhlH was expressed and purified, as described in our previous study (28). The *phlF* gene was amplified by PCR from *P. fluorescens* 2P24 (GenBank accession number DQ083928) and cloned into the modified pET28a vector (Novagene), which contains an N-terminal 6× His tag. The cloning junctions were confirmed by DNA sequencing. Each recombinant plasmid was transformed into the *E. coli* Rosetta (DE3) strain (Novagen). Cells were grown at 37°C in LB medium containing 100 mg/L kanamycin and 40 mg/L chloramphenicol. Expression of PhlF was induced at an OD_600_ of 0.6 by adding 0.2 mM isopropyl-β-D-1-thiogalactopyranoside followed by incubation at 16°C for 20 h. The cells were harvested and sonicated in 20 mM Tris–HCl buffer, pH 8.0, containing 100 mM NaCl in an ice-water bath. After centrifugation, the His-tagged fusion proteins were isolated with Ni-NTA affinity column (GE Healthcare) and purified by gel filtration (Superdex 75, GE Healthcare) in a buffer containing 20 mM Tris–HCl, pH 8.0, with 100 mM NaCl. The eluted proteins were collected and concentrated using centrifugal ultrafiltration for further study.

### Electrophoretic mobility shift assay

6-Carboxyfluoresce in-labeled DNA fragments were obtained by annealing primers. The DNA fragments were added at a concentration of 0.2 µM and incubated at room temperature for 10 min with protein PhlH and PhlF in a buffer containing 0 mM Tris–HCl at pH 8.0, 0.5 mM KCl, 6 mM MgCl_2_, 0.1% (vol/vol) glycerol, 2 mM EDTA, and 1 mM dithiothreitol and 3 µM of human serum albumin in a total volume of 20 µL. Reactions were prepared by adding PLT to a final concentration of 0–500 µM or 0–1 mM. After incubation for 30 min at 25°C, the mixtures were directly subjected to 6% native PAGE with 1× Tris–acetate–EDTA buffer. Electrophoresis was performed at 80 V, 4°C, in an ice-cold bath. For 6-carboxyfluorescein-labeled probes, the images were collected and analyzed on a Typhoon FLA-9500 imaging system (GE Healthcare).

### Isothermal titration calorimetry

Isothermal titration calorimetry (ITC) experiments were performed at 25°C, employing a MicroCal iTC200 instrument (GE Healthcare). The proteins (PhlH and PhlF) and ligands (2,4-DAPG and PLT) were dissolved in a buffer containing 20 mM Tris–HCl, pH 8.0, 100 mM NaCl, and 0.5% (vol/vol) dimethyl sulfoxide (DMSO) and degassed before use. The sample cell was loaded with 220 µL protein samples PhlH at 20 μM or PhlF at 50 μM, whereas the injection syringe was loaded with 40 µL PLT at 0.3 or 0.75 mM. An initial 0.4 µL injection, which was subsequently removed during data analysis, was followed by 19 injections of 2.0 µL each. These injections were spaced at 2 min intervals. Additionally, heats of dilution were determined by titrating the PLT into a solution buffer (20 mM Tris–HCl, pH 8.0, 100 mM NaCl, 0.5% (vol/vol) DMSO and subtracted from the raw titration data. Data analysis was performed with the MicroCal Origin software accompanying the ITC instrument.

### Construction of transcriptional enhanced green fluorescent protein (EGFP) fusion and luminescence dose–response microplate assay

The upstream *phlG* gene promoter P*phlG* (147 bp) and the upstream *phlA* gene promoter P*phlA* (417 bp) were amplified from *P. fluorescens* 2P24 genomic DNA by PCR. Purified PCR products were digested with appropriate restriction enzymes and cloned ahead of a promoterless EGFP gene at the pSEVA225T-derived vector pSEVA225T-EGFP, respectively. The derived vector pSEVA225T-EGFP contains a promoter-free EGFP gene, which can be used to characterize the expression level of the target gene. After verification by sequencing, the constructed plasmid was transferred to the original *P. fluorescens* 2P24 and the corresponding deletion strain by shuttling strain *E. coli* S17-1, and the fluorescent reporter strain fused with EGFP was obtained (2P24::pSEVA225T-EGFP-P*phlG*, 2P24::pSEVA225T-EGFP-P*phlA*, ∆*phlH*::pSEVA225T-EGFP-P*phlG*, ∆*phlF*::pSEVA225T-EGFP-P*phlA*). The fused strains transformed into plasmids were grown to stationary phase at 28°C at 200 rpm in sterilized LB broth, then inoculated into sterilized KB medium in the presence or absence of 20 μM PLT and incubated for 7 h to a cell turbidity of 0.8 at 600 nm. Cultured bacterial solution (200 μL) was taken as the sample, and the luminescence of the sample was measured by a Varioskan Flash Spectral Scanning Multimode Reader (Thermo Scientific) at excitation and emission wavelengths of 485 and 510 nm and normalized to cell density. The software GraphPad Prism version 8 was used for data analysis and drawing. Assays were performed in at least three duplicates.

### Phylogenetic tree construction

The phylogenetic tree was constructed using the bootstrap neighbor-joining (NJ) method with a confidence level of 1,000 replicates in MEGA version 11.0 (29). Bootstrap values were indicated at the nodes for robustness. Homologous PhlH and PhlF protein sequences from six *Pseudomona* strains producing 2,4-DAPG were retrieved from the National Center for Biotechnology Information (NCBI) database (https://www.ncbi.nlm.nih.gov) and the Pseudomonas Genome Database (https://www.pseudomonas.com/).

### Statistics

Statistical analyses were performed using GraphPad Prism version 10.2.1. A two-way analysis of variance with multiple comparison test was used to assess significance among all groups. For comparisons between two means, a two-tailed Student’s *t*-test was applied. Unless otherwise stated, data are presented as mean ± standard deviation.

## RESULTS

### PLT contributes to the delayed growth of non-PLT-producing *P. fluorescens* 2P24 and inhibits the 2,4-DAPG production

Considering the limited research on the effects of environmental factors and nutrients on PLT production, it is difficult to fully determine the exact concentration of PLT synthesized in the environment. *Pseudomonas* PA1201 produces PLT at its highest concentration, reaching 24.5 mg/(OD_600_·L) at 24 h post-inoculation (hpi) and 48.3 mg/(OD_600_·L) at 48 hpi in nutrient-poor minimal medium, equivalent to 90 µM/OD_600_ and 177 µM/OD_600_, respectively (30) In comparison, the well-studied PLT-producing strain *Pseudomonas protegens* Pf-5 can produce PLT at concentrations as high as 0.635 mg/(OD_600_·L) in NBGly medium, equivalent to 2.3 µM/OD_600_ (24). Although the levels of PLT vary significantly between different strains, it still suggests that PLT-producing *Pseudomonas* strains may synthesize high levels of PLT in close proximity to other bacteria, with production influenced by environmental conditions and nutrient availability.

**Fig 1:**
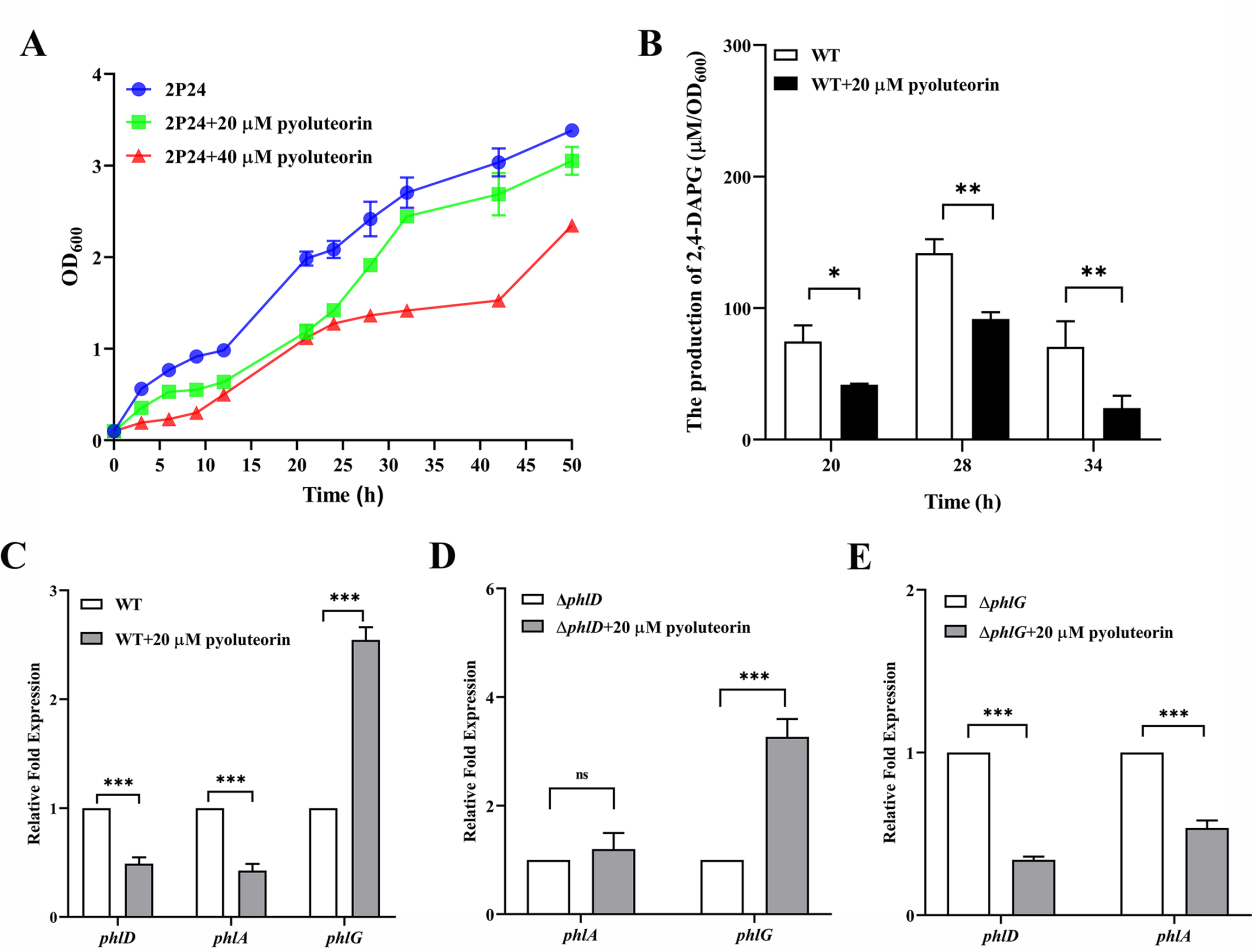
Effect of PLT on growth and 2,4-DAPG production of *P. fluorescens* 2P24 by regulating the transcription of 2,4-DAPG-related genes. (**A**) Effects of 20 and 50 µM PLT on the cell growth of *P. fluorescens* 2P24. The initial cell turbidity was 0.1 at 600 nm. (**B**) HPLC quantitative analysis of 2,4-DAPG in *P. fluorescens* 2P24 after the addition of 20 µM PLT. (**C**) Relative expression levels of *phlA*, *phlD*, and *phlG* were quantified by RT-qPCR using RNA extracted from *P. fluorescens* 2P24 in the absence or presence of 20 µM PLT, respectively, at 18 h post-inoculation. (**D**) Relative expression levels of *phlA* and *phlG* were quantified by RT-qPCR using RNA extracted from 2P24Δ*phlD* in the absence or presence of 20 µM PLT, respectively, at 18 h post-inoculation. (**E**) Relative expression levels of *phlA* and *phlD* were quantified by RT-qPCR using RNA extracted from 2P24Δ*phlG* in the absence or presence of 20 µM PLT, respectively, at 18 h post-inoculation. Error bars denote standard deviations of three independent replicates (*n* = 3). Statistical analyses were performed using *t*-test and two-way analysis of variance. *P < 0.05; **P < 0.01; ***P < 0.001. ns, non-significant; RT-qPCR, reverse transcription quantitative PCR; WT, wild type.

To assess the effects of PLT on bacterial physiology, we compared the growth characteristics of non-PLT-producing *P. fluorescens* 2P24 with and without 20 or 40 µM PLT. Our findings indicated that the presence of 20 µM PLT caused an initial suppression of the growth of *P. fluorescens* 2P24 during the lag phase. Furthermore, increasing the concentration of PLT to 40 µM resulted in a marked reduction in the growth starting from the exponential phase (Fig. 1A). The growth of *P. fluorescens* 2P24 exhibited a negative correlation with PLT, suggesting that subinhibitory levels of PLT suppressed bacterial growth and potentially affected its metabolic function. In PLT- and 2,4-DAPG-producing strain *P. protegens* Pf-5, transcriptional repressor PhlH from *P. protegens* Pf-5 (Pf-5_PhlH) binds to PLT to negatively regulate the biosynthesis of 2,4-DAPG (25). We further investigated the impact of PLT on 2,4-DAPG production by *P. fluorescens* 2P24. HPLC analysis revealed that treatment with 20 µM PLT consistently resulted in a significant reduction in 2,4-DAPG production, with up to a 50% decrease compared to *P. fluorescens* 2P24 without PLT treatment (Fig. 1B). These results suggest that even at low concentrations, PLT could substantially impede the biosynthesis of 2,4-DAPG in *P. fluorescens* 2P24.

### PLT alters 2,4-DAPG autoinduced signaling pathways by regulating the transcription of 2,4-DAPG-related genes

We treated *P. fluorescens* 2P24 with 20 µM PLT and subsequently utilized RT-qPCR to quantify the relative expression levels of *phlA*, *phlD*, and *phlG*. Compared to untreated *P. fluorescens* 2P24, the transcription levels of *phlA* and *phlD* decreased by more than half, while the transcription level of the *phlG* showed a onefold increase following 20 µM PLT treatment (Fig. 1C). Since PhlH typically derepresses the expression of PhlG under high levels of 2,4-DAPG, we speculated that the regulation of *phlG* expression by PLT may depend on PhlH, and PLT may induce the negative transcriptional regulation of *phlA* and *phlD* through alternative mechanisms distinct from the 2,4-DAPG autoinduction mediated by binding transcriptional repressor PhlF.

Considering the potential simultaneous effects of 2,4-DAPG and PLT on the transcription of *phl* genes, we then utilized the Δ*phlD* gene deletion mutant of *P. fluorescens* 2P24 (2P24Δ*phlD*), rendering the bacteria deficient in 2,4-DAPG synthesis. The 2P24Δ*phlD* mutant was confirmed to be deficient in 2,4-DAPG production. The transcriptional analysis of *phlG* was performed by RT-qPCR in the 2P24Δ*phlD* mutant with or without PLT. These results showed that the transcript levels of the *phlG* were significantly increased in 20 µM PLT compared to the control without PLT (Fig. 1D). This demonstrated that the regulatory mechanism governing *phlG* gene expression is mediated by PLT.

To further investigate the potential influence of the low levels of 2,4-DAPG on decreased transcription of *phlA* and *phlD* by affecting PhlF, we generated the Δ*phlG* gene deletion mutant of *P. fluorescens* 2P24 (2P24Δ*phlG*) with the increased production of 2,4-DAPG identified by HPLC. Further analysis of *phlA* and *phlD* transcription in 2P24Δ*phlG* mutant treated with or without 20 µM PLT showed that PLT significantly inhibited *phlA* and *phlD* in the 2P24Δ*phlG* mutant (Fig. 1E). This suggests that PLT is involved in the down-regulation of the *phlACB* operon. We also examined *phlA* and *phlD* expression in a *phlF* deletion mutant (2P24Δ*phlF*), with or without PLT treatment. The results indicated that deletion of *phlF* did not affect the expression levels of *phlA* and *phlD* under these conditions (Fig. S1). Overall, these findings imply that PLT affects the expression of *phl* genes in *P. fluorescens* 2P24 through both 2,4-DAPG-independent signaling pathways, highlighting the exogenous PLT signaling regulatory network governing 2,4-DAPG production in this bacterium.

### Transcription regulators PhlF and PhlH can bind PLT to regulate the *phl* biosynthesis gene cluster

**Fig 2:**
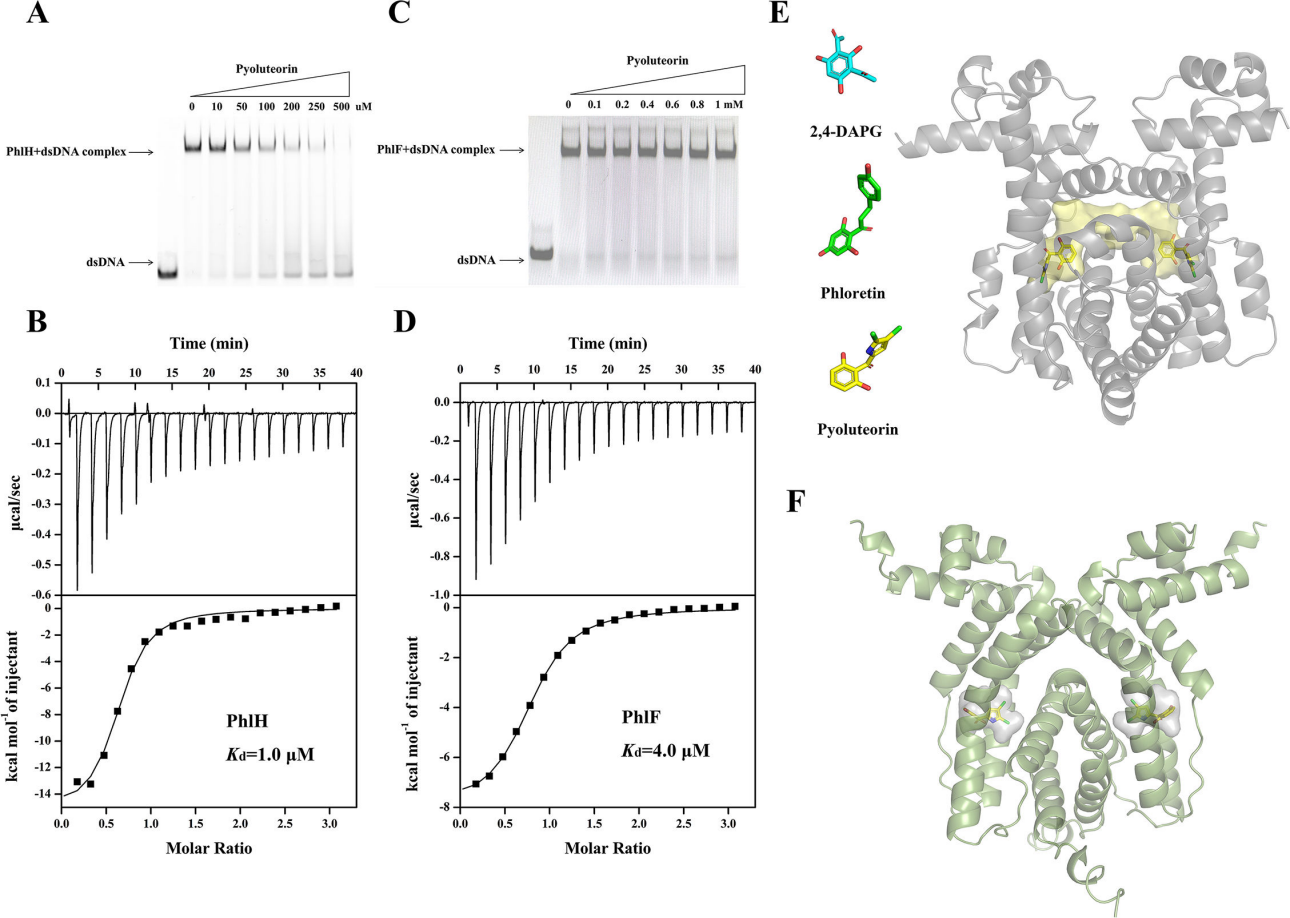
Transcription regulators PhlF and PhlH can bind PLT for regulating the *phl* biosynthesis gene cluster. (**A and C**) Electrophoretic mobility shift assays of 2P24_PhlH (**A**) or 2P24_PhlF (**C**) with the upstream region of *phlG* (free DNA) in the increasing concentrations (0–500 µM) of PLT or with the upstream region of *phlG* (free DNA) in the rising concentrations (0–1 mM) of PLT, respectively. (**B and D**). The binding affinities of 2P24_PhlH (**B**) or 2P24_PhlF (**D**) with PLT were evaluated using ITC analysis. The binding curves corrected for the dilution effects were fit to a one-site binding model, and the *K*_*d*_ values were calculated by the NanoAnalyze software. (**E**) Cartoon representation of 2P24_PhlH in complex with the docked PLT. 2P24_PhlH is in light gray. The ligand-binding tunnel is the hydrophobic tunnel in ligand recognition and is yellow as a transparent surface. 2,4-DAPG, phloretin, and PLT are shown as sticks in cyan, green, and yellow, respectively. (**F**) 2P24_PhlF model calculated using AlphaFold and cartoon representation of model 2P24_PhlF in complex with the docked PLT. Model 2P24_PhlF is in light green. The ligand-binding pocket is predicted ligand recognition and is light gray as a transparent surface. The bound docked PLT is shown as sticks in green. All structure figures were prepared with PyMOL.

The PhlH from *P. fluorescens* 2P24 (2P24_PhlH) shows 69% sequence identity with Pf-5_PhlH. We deduced that 2P24_PhlH could recognize heterologous PLT to induce the expression of *phlG*. Electrophoretic mobility shift assays (EMSAs) were performed to monitor if PLT induces the dissociation of purified His6-tagged PhlH and the 30 bp DNA fragment of the promoter region of *phlG* (*P*phlG). PhlH binds to the *phlG* operator sequence between *phlH* and *phlG*, which is released from DNA with an increased PLT concentration (Fig. 2A). The structure of 2P24_PhlH in its 2,4-DAPG-bound form reveals a hydrophobic tunnel-like cavity that confers promiscuity for binding hydrophobic ligands such as 2,4-DAPG and plant flavonoid phloretin (28). PLT shares a similar phloroglucinol moiety with 2,4-DAPG and phloretin, which can form a similar mode of interaction with PhlH by engaging in π–π stacking with the aromatic amino acid Phe. Molecular docking analysis revealed that PLT occupies a similar binding site as 2,4-DAPG in 2P24_PhlH (Fig. 2E). ITC confirmed that 2P24_PhlH has a high binding affinity for PLT, with a dissociation constant (*K*_*d*_) of 1 µM, which is much smaller than the dissociation constant for the 2P24_PhlH–2,4-DAPG interaction (Table 1; Fig. 2B). This implies that 2,4-DAPG and PLT compete for binding to 2P24_PhlH, with PLT exhibiting higher affinity.

**TABLE 1:** Thermodynamic parameters obtained by isothermal titration calorimetry for TetR-type repressors PhlH and PhlF binding to PLT or 2,4-DAPG

Protein	Ligand	*K_a_* (Μ^−1^)	*K_d_* (μΜ)	Δ*G* (kcal/mol)	Δ*H* (kcal/mol)	*T*Δ*S* (kcal/mol)
PhlH	PLT	9.75 × 10^5^	1.0	−8.5	−15.3	−7.2
PhlH^*a*^	2,4-DAPG^*a*^	1.17 × 10^5^^*a*^	8.5^*a*^	−6.8^*a*^	−12.2^*a*^	−5.4^*a*^
PhlF	PLT	2.47 × 10^5^	4.0	−7.3	−8.0	−0.7

^*a*^ Data from reference (26).

The transcription levels of *phlA* and *phlD* were decreased by heterologous PLT, and PhlF as repressor down-regulate expression of *phlA* and *phlD* in *P. fluorescens* 2P24. EMSAs were conducted to check for the effect of PLT on the dissociation of purified His6-tagged PhlF from the operator DNA sequence between *phlF* and *phlA* (Fig. 2C). Although 2P24_PhlF shares only 20% of sequence identity with 2P24_PhlH, it has a high similarity with the ligand-binding site, especially conserved residue Phe for binding phloroglucinol moiety of ligands via π–π stacking. Structural modeling and docking of 2P24_PhlF, predicted using AlphaFold (31) and AutoDock Vina (32), revealed a similar hydrophobic pocket with conserved residue Phe to bind PLT (Fig. 2F). ITC experiments showed that 2P24_PhlF can bind to PLT with a *K*_*d*_ of 4 μM (Table 1; Fig. 2D). Interestingly, PLT does not dissociate PhlF from the DNA but exhibits a significantly high affinity for PhlF, indicating that PLT acts as an inhibitor targeting PhlF, potentially preventing PhlF dissociation from DNA and enhancing the repression of *phlD* and *phlA* transcription.

### PLT is required for transcription regulators PhlF and PhlH to impact the *phl* biosynthesis gene expression *in vivo*

**Fig 3:**
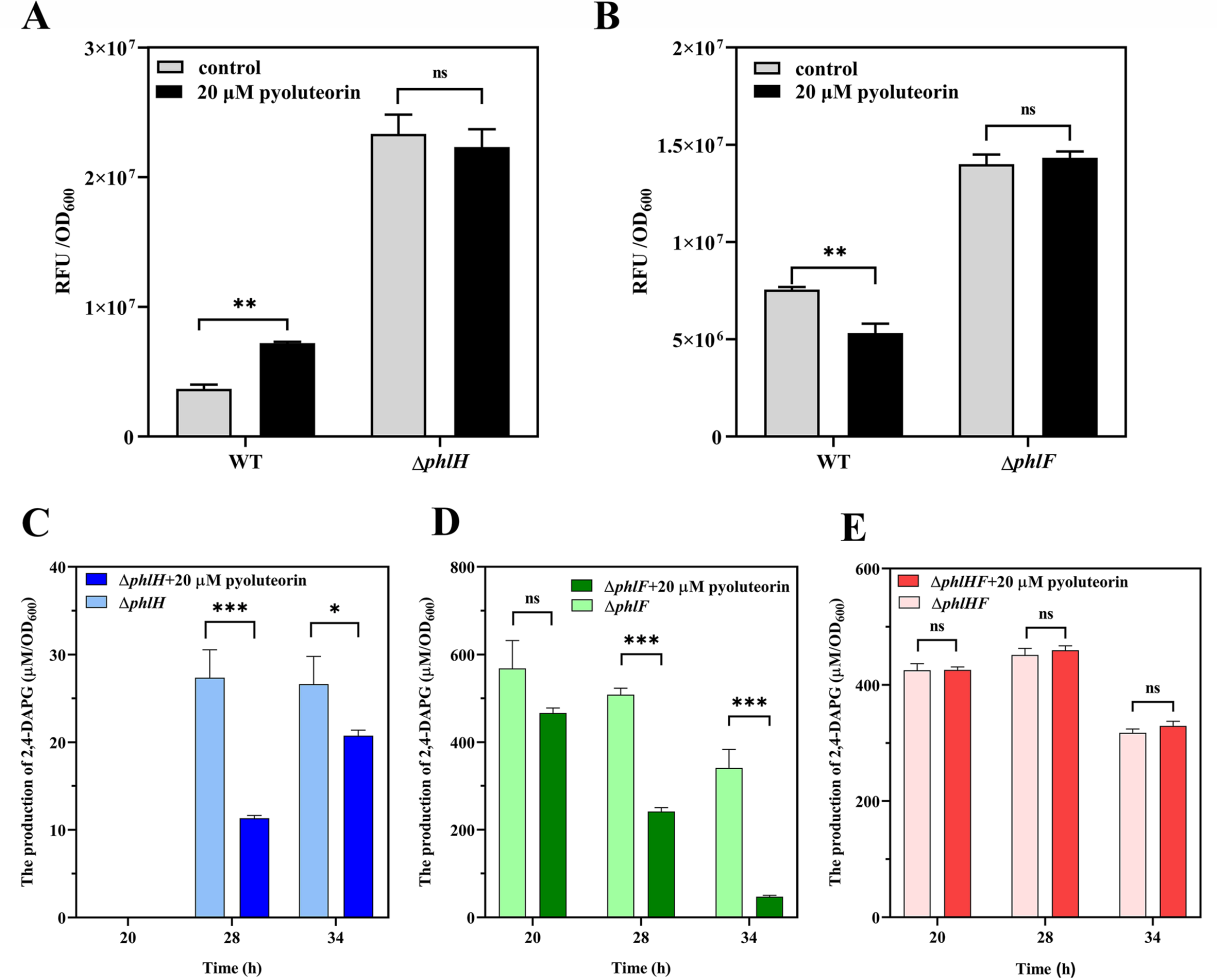
In response to PLT, PhlH regulates the expression of *phlG*, while PhlF regulates the expression of *phlA*. (**A**) The fluorescent activity of the pSEVA225T-EGFP-P*phlG* plasmid was evaluated in WT and Δ*phlH* strains to test the expression of *phlG*. The bars illustrate the relative fluorescent units (RFU) normalized to a 1 mL culture with OD_600_ = 1. (**B**) The fluorescent activity of the pSEVA225T-EGFP-P*phlA* plasmid was evaluated in WT and Δ*phlF* strains to test the expression of *phlA*. (**C–E**) 2,4-DAPG production of 2P24Δ*phlH* (**C**), 2P24Δ*phlF* (**D**), and 2P24Δ*phlH*Δ*phlF* (**E**) strains grown in KB medium with or without 20 µM PLT. Error bars denote standard deviations of three independent replicates (*n* = 3). Statistical analyses were performed using *t*-test and two-way analysis of variance. *P < 0.05, **P < 0.01, ***P < 0.001. ns, non-significant.

To confirm the roles of PhlH and PhlF in regulating the *in vivo* expression of *phlG* and *phlA* in response to exogenous PLT, we constructed PhlH or PhlF deletion mutants using *P. fluorescens* 2P24 as the parental strain for subsequent promoter-EGFP analysis. We then measured the EGFP fluorescence of the mutants and their parental strain in the presence or absence of PLT. Upon the addition of 20 µM PLT, the *phlG*-EGFP reporter activity was increased in the wild-type strain, but no significant changes were observed in the Δ*phlH* strain (Fig. 3A). This indicates that the induction of *phlG* expression by PLT was dependent on PhlH. Conversely, the *phlA*-EGFP reporter activity was decreased in the wild-type strain, but no significant changes were observed in the Δ*phlF* strain (Fig. 3B), indicating that the repression of *phlA* expression by PLT was dependent on PhlF.

Further analysis using HPLC confirmed a significant reduction in 2,4-DAPG production in the 2P24Δ*phlH* mutant, nearing 30 µM, with even lower levels observed after treatment with 20 µM PLT. This suggests that PhlF continues to repress *phl* biosynthesis gene transcription by sensing PLT (Fig. 3C). Similarly, HPLC analysis showed a significant increase in 2,4-DAPG in the 2P24Δ*phlF* mutant, reaching approximately 600 µM. Upon treatment with 20 µM PLT, the levels of 2,4-DAPG decreased further in the 2P24Δ*phlF* mutant, indicating that PhlH derepresses *phlG* transcriptions by sensing PLT (Fig. 3D). Further investigation using HPLC analysis to determine 2,4-DAPG production in the 2P24Δ*phlH*Δ*phlF* double mutant treated with or without 20 µM PLT revealed no significant changes (Fig. 3E). This demonstrates that PhlH and PhlF are the main regulators impacting *phl* biosynthesis gene expression by sensing exogenous PLT.

### Phylogenetic analysis of 2,4-DAPG gene clusters and homolog identification of its transcriptional factor in signaling

**Fig 4:**
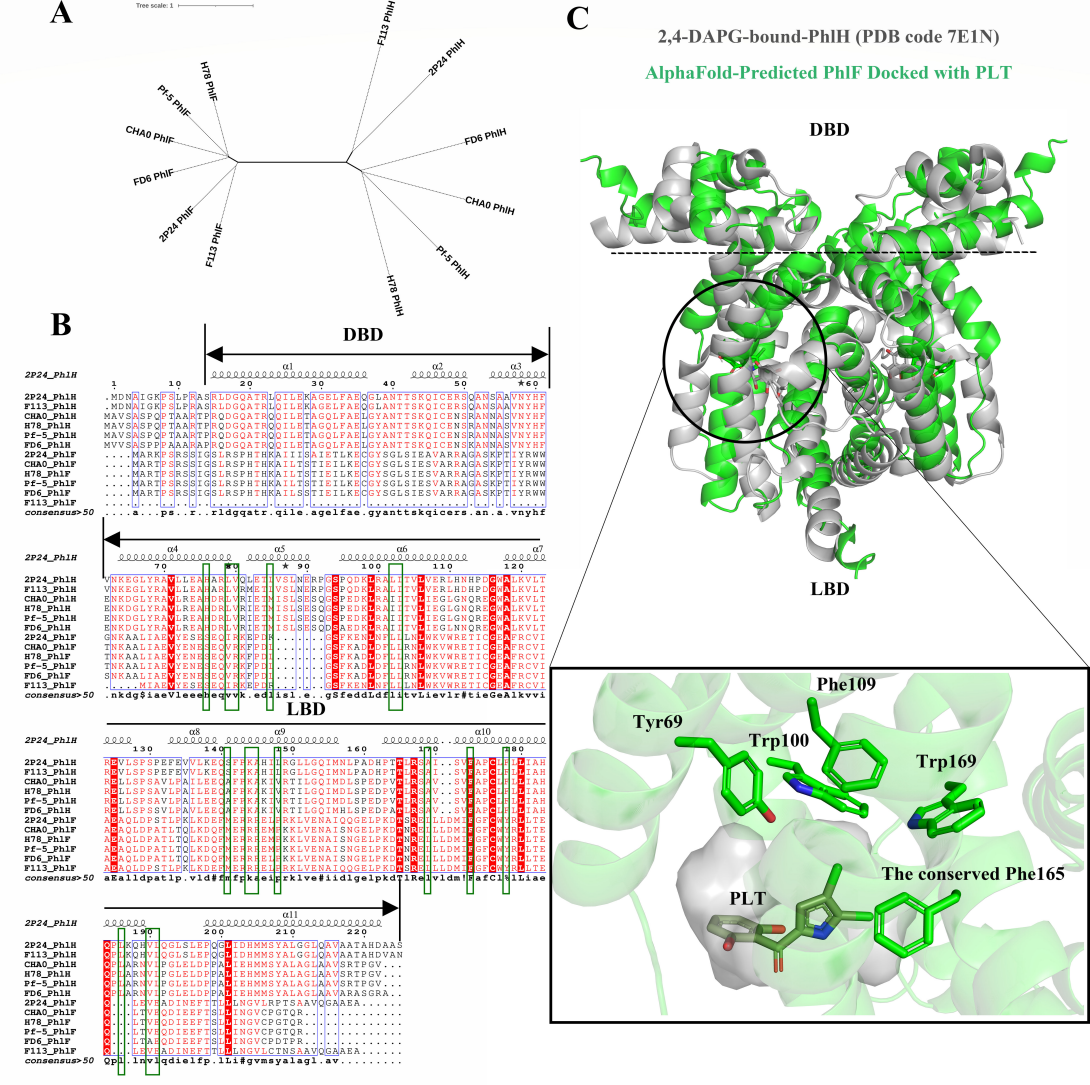
The PhlF and PhlH share similarities with the ligand-binding domain (LBD). (**A**) Phylogenetic tree of TetR-type repressors PhlH and PhlF in representative strains form *Pseudomonas* spp. constructed using the neighbor-joining method. (**B**) Multiple sequence alignment of PhlH and PhlF proteins from representative *Pseudomonas* spp. Residues involved in binding 2,4-DAPG are shown in green boxes. The alignment is performed using MultAlin (33) and ESPript (34). The secondary structural elements of 2P24_PhlH are displayed at the top of the alignment. (**C**) The superposition of 2,4-DAPG-bound PhlH (PDB code 7E1N) (gray) and model PLT-bound PhlF (green) dimers is shown in the cartoon representation (top). The N-terminal DNA-binding domain (DBD) comprises three helices, with helices α2 and α3 forming the conventional helix-turn-helix-DNA-binding motif. The LBD is composed of the remaining helices. 2,4-DAPG and the bound docked PLT are shown as sticks in gray and green, respectively. A close-up view of the interaction mode between the AlphaFold-predicted PhlF (shown in transparent green) and the docked PLT (shown in dark green) is displayed at the bottom. The PLT-binding pocket, along with aromatic residues in the vicinity of PLT (highlighted in green), is shown as a stick, illustrating the potential interactions involved in the ligand-binding process. All structure figures were prepared with PyMOL.

The metabolic co-regulation between 2,4-DAPG and PLT biosynthetic pathways has been identified in strains of *P. protegens* Pf-5 (14, 25). Besides acting as biocontrol agents against different pathogens, 2,4-DAPG and PLT are significant for intraspecies signaling and cross-regulation in *P. protegens* Pf-5. TetR-type repressors PhlH and PhlF play a crucial role in recognizing signals to regulate 2,4-DAPG biosynthesis. To understand the conservation of *phl* gene clusters, we compared PhlH and PhlF complete genomes available on the NCBI database. We used a *phl* gene cluster (*phlHGFACBD*) of *P. protegens* Pf-5 as a query to search genomic databases and classified them into two major groups. One includes both PLT and 2,4-DAPG-producing *Pseudomonas protegens* strains like *P. protegens* Pf-5, CHA0, H78, and FD6, while the other comprises only 2,4-DAPG-producing strains like *P. fluorescens* 2P24 and F113 (Fig. 4A).

To explore the functions of these regulators in sensing signals, we analyzed the homology of PhlH and PhlF from representative strains *P. protegens* (Pf-5, CHA0, H78, and FD6) and *P. fluorescens* (2P24 and F113) from two groups. Using the identified PhlH and PhlF proteins, we constructed a phylogenetic tree with a bootstrap value with 1,000 replicates through NJ. These analyses revealed a common consensus for PhlH and PhlF, consistent with the classification group from *phl* gene cluster comparison. Although PhlH had low sequence similarity to PhlF, both belong to the TetR-family, featuring N-terminal DNA-binding domains (DBDs) with a helix-turn-helix motif and C-terminal LBDs. Multiple alignments of PhlH and PhlF from the two groups revealed that its ligand-binding domains are more conserved than their DNA-binding domains, indicating that the ligand-binding domains from PhlH and PhlF are evolutionarily related (Fig. 4B). Interestingly, both PhlH and PhlF from *P. fluorescens* 2P24 can sense 2,4-DAPG, suggesting similar ligand-binding sites. Superposition of 2,4-DAPG-bound PhlH and model PLT-bound PhlF dimers from *P. fluorescens* 2P24 revealed that the LBD showed a similar interior pocket for ligand binding, surrounded by helices. The Phe165 residue from PhlF is conserved with Phe173 in PhlH from *P. fluorescens* 2P24, which plays a crucial role in facilitating the binding of the phloroglucinol moiety of 2,4-DAPG through π–π stacking interactions. Additionally, aromatic residues adjacent to the PLT-binding pocket, such as Tyr69, Trp100, Phe109, and Trp169 in PhlF, may also contribute significantly to binding, as predicted by the AlphaFold model (Fig. 4C). This suggests that both PhlH and PhlF in *Pseudomonas* spp. may share a similar mechanism for the recognition and binding of PLT to sense the signal molecule. However, while PLT binds directly to PhlF, it does not induce dissociation of PhlF from DNA. This suggests that PLT binding does not trigger the typical structural rearrangements in molecular switches of PhlF that are generally involved in the allosteric regulation of transcription for the *phl* gene cluster. These findings imply that PhlF may employ an atypical allosteric switching mechanism.

## DISCUSSION

Rhizosphere microorganisms have evolved the capability to produce and release signals that play crucial roles in shaping microbial communities, influencing both microbial competition and cooperation (2). Among these signals, antibiotics at low and non-inhibitory concentrations serve as intra- and interspecies signals, modulating microbial growth and activity (7). However, the precise mechanisms by which these antibiotics act as interspecies signals to coordinate gene expression remain largely unexplored. In our study, we elucidate the role of exogenous PLT as a key component in an interspecies signaling pathway that regulates the expression of *phl* biosynthesis genes for 2,4-DAPG production, which are integral to biological activity. Interestingly, although PLT and 2,4-DAPG have been identified as biological agents against plant pathogens, there appears to be cross-regulation between these compounds in *Pseudomonas* strains, indicating a complex interplay among rhizosphere microbial species with implications for plant health and disease resistance.

Antibiotics are effective against competitors only above a certain threshold concentration, requiring a sufficiently high density of cells (35). Moreover, the production of antibiotics, which can impose metabolic costs on cooperation, is closely related to bacterial community stability within environmental conditions (36). Polyketide antibiotics, 2,4-DAPG and PLT, are produced by a wide range of *Pseudomonas* spp. in soil and rhizosphere environments (10). 2,4-DAPG is biosynthesized through the decarboxylative condensation of malonyl-CoA-derived extender units in a process similar to fatty acid synthesis (37). On the other hand, the hybrid polyketide-non-ribosomal peptide molecule PLT involved condensation of malonyl-CoA to generate a resorcinol ring with chlorinated pyrrole moiety (38). Interestingly, these two biosynthetic pathways may compete for utilizing malonyl-CoA, potentially impacting the production of these polyketide antibiotics. It presents extensive cross-regulation between the synthesis of 2,4-DAPG and PLT in *P. protegens* Pf-5. The 2,4-DAPG biosynthetic intermediate chlorinated phloroglucinol can function as a signal regulating PLT production (14). Moreover, in response to 2,4-DAPG, the PLT biosynthetic regulator PltZ homolog from *Pseudomonas aeruginosa* American Type Culture Collection 27853 can induce a putative ABC transporter and enhance antibiotic resistance (39). The biosynthesis of 2,4-DAPG is negatively regulated by PhlH sensing to PLT in *P. protegen* Pf-5 (25). The sequential signaling pathway between *P. protegens* and *P. capeferrum* shows that a high level of PLT induced by *P. capeferrum* results in the lysis of *P. capeferrum* and subsequently inhibits 2,4-DAPG production of *P. protegens* (26). Taken together, these works show that interspecies signaling between 2,4-DAPG and PLT influences biological control activity and bacterial communication in soil and rhizosphere environments.

Considering the perspective of these antibiotics as intraspecies signaling, we propose that PLT can act as an interspecies signal to manipulate non-PLT but 2,4-DAPG-producing *Pseudomonas* strains, thereby facilitating communication among microorganisms in the rhizosphere. *P. fluorescens* 2P24, isolated from wheat rhizosphere in take-all decline soil, produces antimicrobial metabolite 2,4-DAPG (40). This compound exhibits broad-spectrum antifungal activities, effectively suppressing soil-borne pathogens such as *Fusarium oxysporum*, *Septoria tritici*, *Thielaviopsis basicola*, and *Rhizoctonia solani* (41). PhlH and PhlF belong to the TetR family, and both act as transcriptional repressors of the 2,4-DAPG biosynthetic genes. PhlF repression is relieved by 2,4-DAPG, leading to positive feedback regulation of 2,4-DAPG biosynthesis (22). Conversely, PhlH, in response to 2,4-DAPG, provides negative-feedback regulation of 2,4-DAPG biosynthesis (23).

The DBD of the TetR family repressor is well known for its role in interacting with the DNA major groove, and its LBD is responsible for binding a wide range of diverse ligands (42). In our previous study, crystal structures of PhlH in both its apo form and 2,4-DAPG-bound form revealed its ligand-recognizing and allosteric switching mechanisms. PhlH harbors a long, hydrophobic ligand-binding tunnel that is essential for ligand-induced DNA dissociation from PhlH, suggesting promiscuity binding modes for diverse ligands such as 2,4-DAPG or several plant-derived flavonoids phloretin (28). Interestingly, the LBD between PhlH and PhlF shows higher sequence similarity than its DBD, implying PhlH and PhlF possibly share a similar ligand-binding pocket. Subsequent analysis shows that PhlF can bind to PLT at the micromolar range and repress the transcription of *phlA*. Structural predictions showed that PhlF presents a hydrophobic inner pocket in its LBD, where PLT molecules can be docked, reminiscent of the crystal structure of 2,4-DAPG-bound PhlH. In addition to PLT, the production of 2,4-DAPG can be inhibited by the mycotoxin fusaric acid from *Fusarium* spp. through an unknown mechanism (43). Moreover, plant-derived flavonoids, including phloretin, participate in the repression of 2,4-DAPG production (44). These studies imply that a wide range of interspecies signals play a crucial role in bacterial metabolic co-regulation of 2,4-DAPG synthesis.

**Fig 5:**
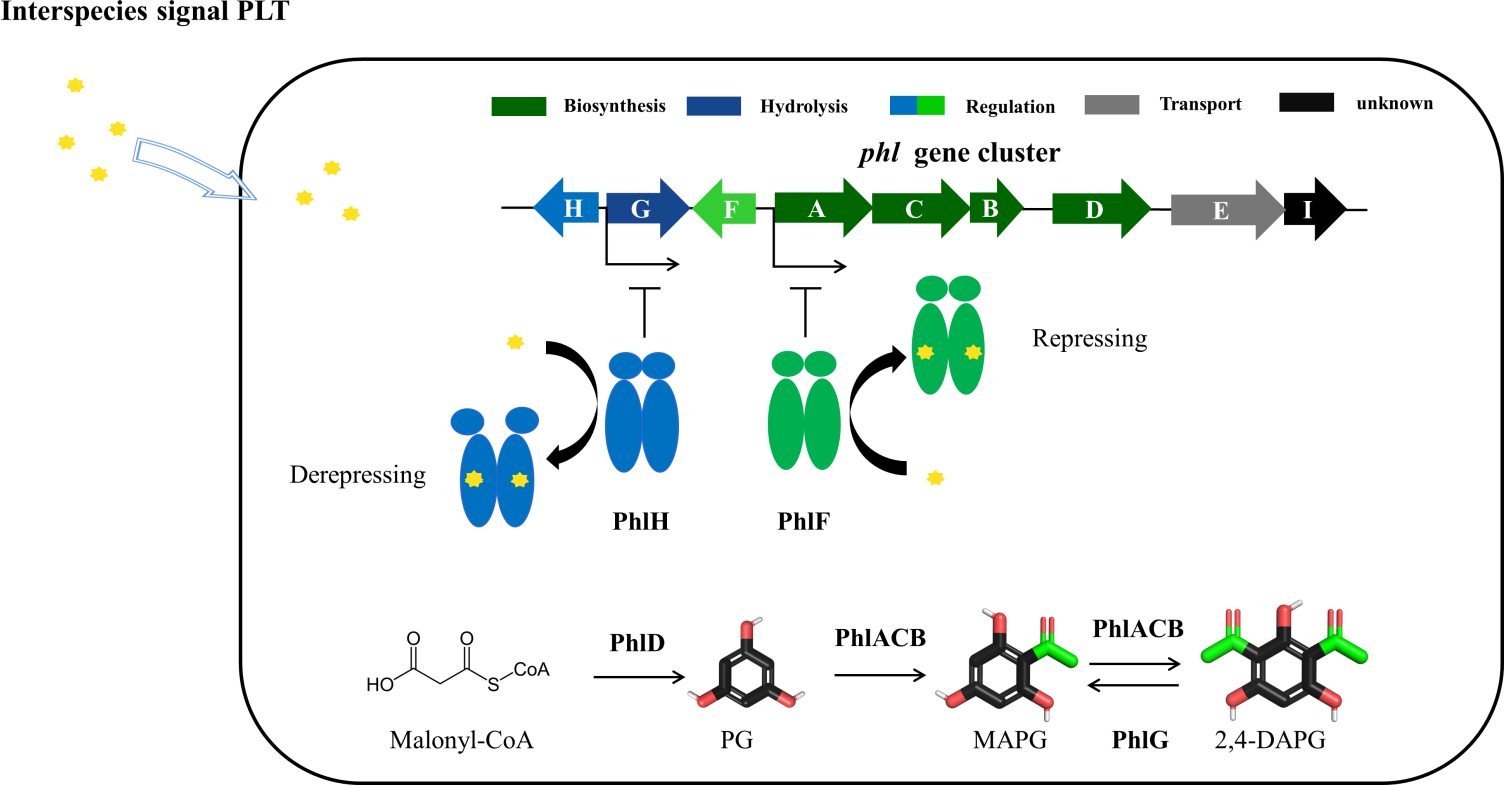
Schematic representation of TetR repressors PhlH and PhlF from the 2,4-DAPG biosynthetic gene cluster positively regulated the hydrolysis of 2,4-DAPG and negatively regulated 2,4-DAPG synthesis in response to interspecies signal PLT.

In summary, our study provides new insights into interspecies signaling, demonstrating how PLT inhibits 2,4-DAPG production in *P. fluorescens* 2P24 by finely tuning the repressors PhlH and PhlF (Fig. 5). This regulatory mechanism effectively manages the trade-off between the costs and benefits of antibiotic production, especially in complex and dynamic environmental conditions. Specifically, the binding of PLT to PhlH leads to the dissociation of PhlH from the promotor region, enabling RNA polymerase to initiate the transcription of the *phlG* gene, which promotes the hydrolysis of 2,4-DAPG. Conversely, PLT binding to PhlF does not induce protein–DNA dissociation from the *phlA promoter*, confirming its role in maintaining a repressed state of PhlF, thereby inhibiting the synthesis of 2,4-DAPG. These findings not only unveil a novel function of the PhlH and PhlF repressors in *P. fluorescens* 2P24, which coordinates secondary metabolic pathways by sensing the interspecies signal PLT, but also suggest a potentially widespread and conserved mechanism among *Pseudomonas* spp. This mechanism, mediated by TetR family regulators, could play a crucial role in modulating microbial competition, survival strategies, and cell–cell communication.

## SUPPLEMENTAL MATERIAL

Supplemental material spectrum.01886-24-s0001.docx Tables S1 and S2; Fig. S1.

ASM does not own the copyrights to Supplemental Material that may be linked to, or accessed through, an article. The authors have granted ASM a non-exclusive, world-wide license to publish the Supplemental Material files. Please contact the corresponding author directly for reuse.
