# Supplementary material for: Interspecies signaling modulates the biosynthesis of antimicrobial secondary metabolites related to biological control activities of Pseudomonas fluorescens 2P24
Source: Microbiol Spectr. 2025 Feb 3;13(3):e01886-24. doi: 10.1128/spectrum.01886-24 (PMC11878095; doi:10.1128/spectrum.01886-24)
Supplement: Supplemental material — Tables S1 and S2; Fig. S1. [file spectrum.01886-24-s0001.docx]

**Supplementary Table S1 Strains and plasmids used in this study.**

| **Strains or plasmids** | **Description** | **Reference or source** |
| --- | --- | --- |
| **Strains** |  |  |
| ***E. coli* strains**  DH5α  BL21 (DE3)  S17-1 λpir | F^–^ *endA1* *glnV44* *thi-1* *recA1* *relA1* *gyrA96* *deoR* *nupG* *purB20* φ80d*lacZ*ΔM15 Δ(*lacZYA-argF*)U169, hsdR17(*r_K_*^–^*m_K_*^+^), λ^–^  pET serial plasmids expression host  F^–^ *ompT gal dcm lon hsdSB(rB^–^mB^–^)* λ(DE3 [*lacI lacUV5-T7p07 ind1 sam7 nin5*]) [*malB^+^*]_K-12_(λS)  F^–^ *thi pro hsdR* [RP4-2 Tc::Mu Km::Tn7 (Tp Sm)] | Novagen  Novagen  Novagen |
| ***Pseudomonas* strains**  *P. fluorescens* 2P24  *ΔphlA*  *ΔphlG*  *ΔphlF*  *ΔphlH*  *ΔphlHF*   1. *protegens Pf-5* | Wild type; 2,4-DAPG+ ; mupironcin+ ; Amp^r^; Cm^r^  *phlA* gene in-frame deletion in strain 2P24; Ap^r^  *phlG* gene in-frame deletion in strain 2P24; Ap^r^  *phlF* gene in-frame deletion in strain 2P24; Ap^r^  *phlH* gene in-frame deletion in strain 2P24; Ap^r^  *phlH* and *phlF* gene double mutant; in-frame deletion in strain 2P24; Ap^r^  Wild type; 2,4-DAPG^+^ ; pyoluteorin^+^ | This study  This study  This study  This study  This study |
| **Plasmids**  pK18mobsacB  pK18mobsacB-*ΔphlA*  pK18mobsacB-*ΔphlG*  pK18mobsacB-*ΔphlF*  pK18mobsacB-*ΔphlH*  pET28a  pET28a-PhlH  pET28a-PhlF  pSEVA225T  pSEVA225T-*EGFP*  pSEVA225T-*EGFP*-P*phlG*  pSEVA225T-*EGFP*-P*phlA* | Widely used gene knockout vector; Kan^r^  pK18mobsacB derivative for generation of *phlA* deletion; Km^r^  pK18mobsacB derivative for generation of *phlG* deletion; Km^r^  pK18mobsacB derivative for generation of *phlF* deletion; Km^r^  pK18mobsacB derivative for generation of *phlH* deletion; Km^r^  Protein overexpression vector; Kan^r^  Plasmid for overexpression of PhlH; Km^r^  Plasmid for overexpression of PhlF; Km^r^  Cloning vector for construction of transcriptional fusion in P. fluorescens; Km^r^  pSEVA225T containing promoterless *EGFP*; Km^r^  pSEVA225T containing a P*phlG-EGFP* transcriptional fusion; Km^r^  pSEVA225T containing a P*phlA-EGFP* transcriptional fusion; Km^r^ | This study  This study  This study  This study  This study  This study  This study  This study  This study |

**Supplementary Table S2 Primers used in this study.**

| **Primers** | **DNA targets** | **Sequences (5’ to 3’)** |
| --- | --- | --- |
| **Primers for qRT-PCR**  rpoD-F  rpoD-R  PhlD-F  PhlD-R  PhlA-F  PhlA-R  PhlG-F  PhlG-R | *rpoD*  *phlD*  *phlA*  *phlG* | CGCCAAGAAGTACACCAACC  GCGACGGTATTCGAACTTGT  ATGTGCTCATCGTCTCCCTGG  TGATACCGCATCGCCGAATAAT  TCCTGGAAATGCTCGGCTGC  TACCAAGGCGTATGCGATCTGG  CATCACGAGCGTCTCAGCGA  CAGATCCGGACAGAAGCCCA |
| **Primers for mutants construction**  F1∆*phlA*  R1∆*phlA*  F2∆*phlA*  R2∆*phlA*  F1∆*phlG*  R1∆*phlG*  F2∆*phlG*  R2∆*phlG*  F1∆*phlH*  R1∆*phlH*  F2∆*phlH*  R2∆*phlH*  F1∆*phlF*  R1∆*phlF*  F2∆*phlF*  R2∆*phlF* | *phlA*  *phlG*  *phlH*  *phlF* | AAAGAATTCCGAAAAGCAATGAAACGGAT  AAATCTAGAGCCCAATTGGCCCTTTACCA  AAATCTAGAAAGACCAACGTTCCGCTGCG  AAAAAGCTTAACGATGTCGTATTCACCTG  AAAGGATCCATCGTCGCGCGCGGCGGCGG  AAATCTAGAGAACTGCTTCTGCATTGGCA  AAATCTAGATTCAATCACCTGGCCAGCAT  AAAAAGCTTACAATGGGTTTATTCTTGTA  CGGAATTCGCCGTGGTCCTGTTGACCCACG  GCTCTAGAATGAGCTATGCCCTTGGCGGCC  AATCTAGAGTTGGCCAACCCCTGCTCGGCA  CCCAAGCTTTTGCTGTATTTTTTCGACAGC  AAAGAATTCCGCACTATCAAGGAAATATC  AAATCTAGACTGCTGAACGGGGTATTGCG  AAATCTAGAGATGCTCAACCCCGAATAAC  AAAAAGCTTGTTGCGGTTGATCGTATCGG |
| **Primers used for physiological experiments**  PhlF-F  PhlF-R  P*phlG*-F  P*phlG*-R  P*phlA*-F  P*phlA*-R  phO-F  phO-R  EGFP-F  EGFP-R | *phlF*  P*phlG*  P*phlA*  *phO*  *EGFP* | CGCCATATGATGGCCCGTAAACCATCTCG  CCGCTCGAGTCAGGCTTCGGCGGCGCCCT  GAGCTCATCTCTTTATAGTTCTAA  GACGATATTTCCTTGATAGTGC  AATTCAAAATCTCCAATATGGCGCTTG  GTGTACATCCTCCAGATTCCGTTCTTT  ATGATACGAAACGTGCCGTATCGTTAAGGT  ACCTTAACGATACGGCACGTTTCGTATCAT  AAAAAGCTTAGGAGGAAAAACATATGGTG  AGCAAGGGCGAGGA  AAAACTAGTTTACTTGTACAGCTCGTCCATG |

**Supplementary figure**

**
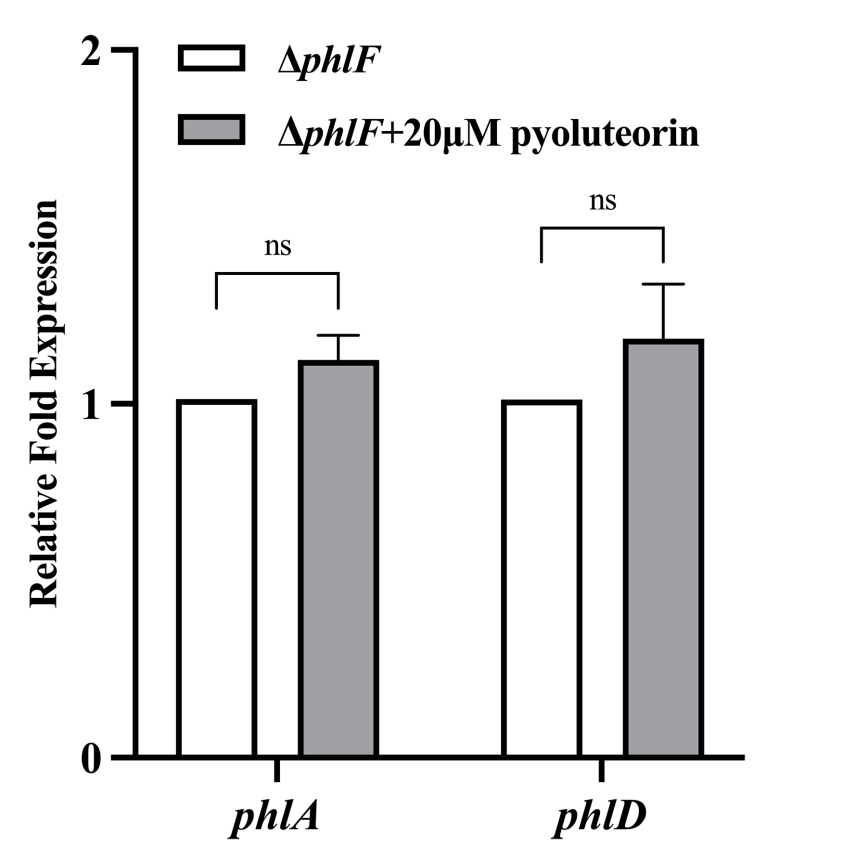
**

Figure S1.

Relative expression levels of *phlA* and *phlD* were quantified by RT-qPCR using RNA extracted from 2P24Δ*phlF* in the absence or presence of 20 μM PLT at 18 h postinoculation. Error bars denote standard deviations of three independent replicates (n = 3). Statistical analyses were performed using the *t* test and two-way ANOVA. ns is non-significant.
